# Supplementary material for: Biology and Physics of Heterochromatin-Like Domains/Complexes
Source: Cells. 2020 Aug 11;9(8):1881. doi: 10.3390/cells9081881 (PMC7465696; doi:10.3390/cells9081881)
Supplement: Supplementary file 1 [file cells-09-01881-s001.pdf]

**Box S1.** Epigenetic compartmental domains (ECDs) in mammals.

Contact enrichments detected in Hi-C maps that are generated by segregation of micro-phase separated heterochromatin-*like* and Pc-G domains/complexes are termed epigenetic compartmental domains (ECDs; [1]). Micro-phase separation of heterochromatin-*like* domains/complexes is caused by "bridging" of H3K9me3-marked nucleosomes by HP1 proteins; micro-phase separation of Pc-G domains/complexes is caused by "bridging" of H3K27me3-marked nucleosomes by Polycomb CBX homologues. Like-with-like segregation of micro-phase separated domains/complexes generates contact enrichments that drive the compartmentalisation detected in Hi-C maps. ECDs overlap with B-type compartmental domains and represent the "epigenetic" component of cellular identity and contribute to safeguarding that identity. The evidence and reasoning that supports this definition of ECDs is detailed below, using as our guide three diagrams.

The conclusion drawn from intersecting data from computational correlation, principle component analysis and ChIP-Seq data was that the cell-type specific checkerboard (or plaid) pattern observed in Hi-C maps represented the folding of chromatin into euchromatin (A-type compartments) and heterochromatin (B-type compartments) [2-4]. Importantly, the H3K9me3 and H3K27me3 epigenetic modifications used to define B-type compartments as heterochromatic are the only histone modifications that are truly epigenetic, *i.e.*, heritable from one cellular generation to the next [5]. This is because each modification has its own *"write-and-read"* mechanism (Diagram **A**; [5]) that enables HP1-containing heterochromatin-*like* and Polycomb-Group (Pc-G; consisting of H3K27me3, PRC1 and PRC2; [6,7]) domains/complexes to be faithfully inherited from one cell generation to the next.

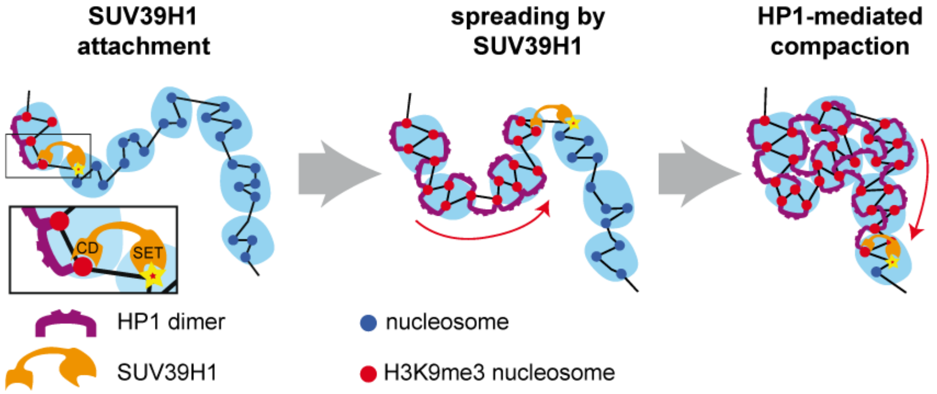


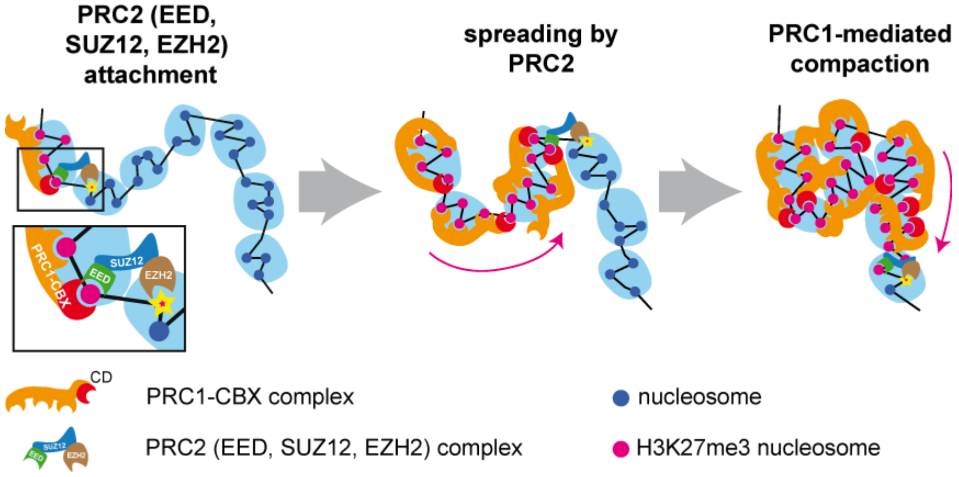

**Diagram A: The *write-and-read* mechanisms for the H3K9me3 and H3K27me3 *epigenetic* histone modifications. Top row:** For the H3K9me3 "write-and-read" mechanism the CD of the SUV39H1 K3K9 HMTase binds H3K9me3 (left), which increases allosteric activation of the SET (methyltransferase) domain that in turn enhances the methylation at H3K9 of neighbouring naïve nucleosomes [8]. The HP1 dimers "bridge" H3K9me3-marked nucleosomes in the wake of the "spreading" H3K9me3 domain, which stabilizes the zig-zag geometry within a "clutch" (middle) with concomitant compaction (right). HP1 bridging of H3K9me3-marked nucleosomes resulting in enrichment (compaction) drives *micro*-phase separation of the heterochromatin-*like* domains/complexes from euchromatin (see Diagram B given next). **Bottom Row:** In the case of H3K27me3, it is the PRC2 complex consisting of the core subunits EED, SUZ12 and EZH2, that regulates the "write-and-read" mechanism. Accordingly, EED binds H3K27me3 and this binding allosterically activates the SET domain of EZH2, which in turn enhances the methylation at H3K27 of neighbouring naïve nucleosomes (left). The CD of a CBX protein that is a homologue of *Drosophila Polycomb* binds in the wake of the "spreading" domain of H3K27me3-marked nucleosomes (middle). The best studied of the mammalian Pc-homologues is CBX2 [9]. CBX2 is a subunit of the PRC1 complex and CBX2 contains a specific domain called the compaction and phase separation (CaPS) domain that compacts (four) nucleosomes [9,10]; the compaction stabilizes the zig-zag geometry within a "clutch" (right). It is the combined action of PRC2-PRC1complexes that drive *micro*-phase separation of Pc-G domains/complexes. The "clutches" were taken and modified from [1].

Like-with-like interactions drive segregation of micro-phase separated heterochromatin-*like* domains/complexes (as shown in Diagram **B**); Pc-G domains/complexes would segregate with Pc-G domains/complexes. Segregation involves both *cis*- (shown in Diagram **B**) and *trans*-interactions (not shown in Diagram **B**).

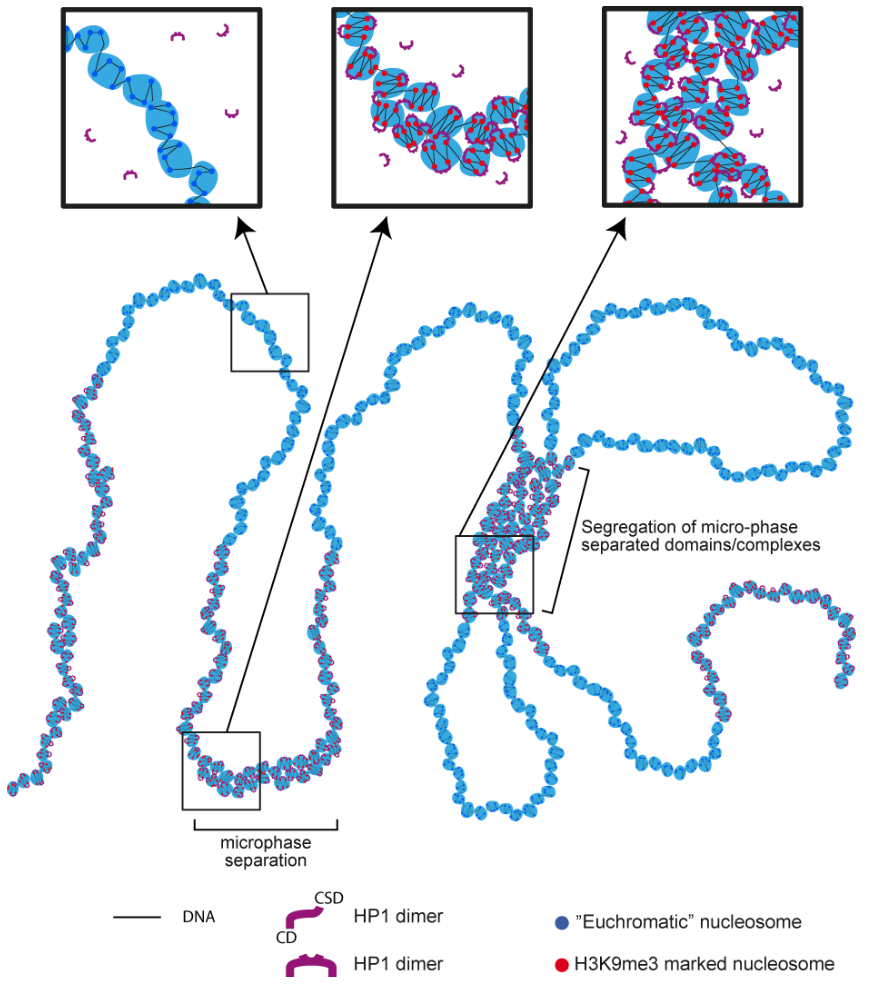

**Diagram B: *Micro*-phase separation and segregation of heterochromatin-*like* domain/complexes.** Bridging of H3K9me3-marked nucleosomes by HP1 results in *micro*-phase separation of heterochromatin-*like* domains/complexes (middle loop). Bridging results in stabilisation of zig-zag geometry of H3K9me3-marked nucleosomes in heterochromatin-*like* "clutches" (middle box at top of figure; modified from [1]). This contrasts with euchromatic "clutches" (box on left) where the nucleosomes are disorganised with only partial zig-zag geometry. Like-with-like attraction (binding potential), due to an entropic effect (see text for details), results in *cis-* (shown in Diagram B) and *trans-* (not shown) interactions between domain/complexes. Interactions are stabilised by bridging of H3K9me3-marked nucleosome fibers by HP1 (box on right) and stabilises segregation of domains/complexes. Contacts resulting from segregation are detected as contact enrichments that contribute to ECDs in Hi-C experiments. Segregation of micro-phase separated domains/complexes is unlikely to be static (as drawn). Micro-phase separated domains/complexes within segregated assemblies will be subject to constant disassociation and association

*Cis-* and *trans-* contacts that result from segregation of heterochromatin-*like* and Pc-G domains/complexes emerge as ECDs in Hi-C maps (Diagram **C**). Notably, screens for genes that are necessary to safeguard cellular identity identified genes that encode CAF-1, the SUMO-conjugating enzyme UBE2i, SUMO2, SETDB1, ATRX and DAXX proteins [11,12]. All are involved in either nucleation or replication of heterochromatin-*like* domains/complexes thus providing a link between safeguarding cellular identity and ECDs [1].

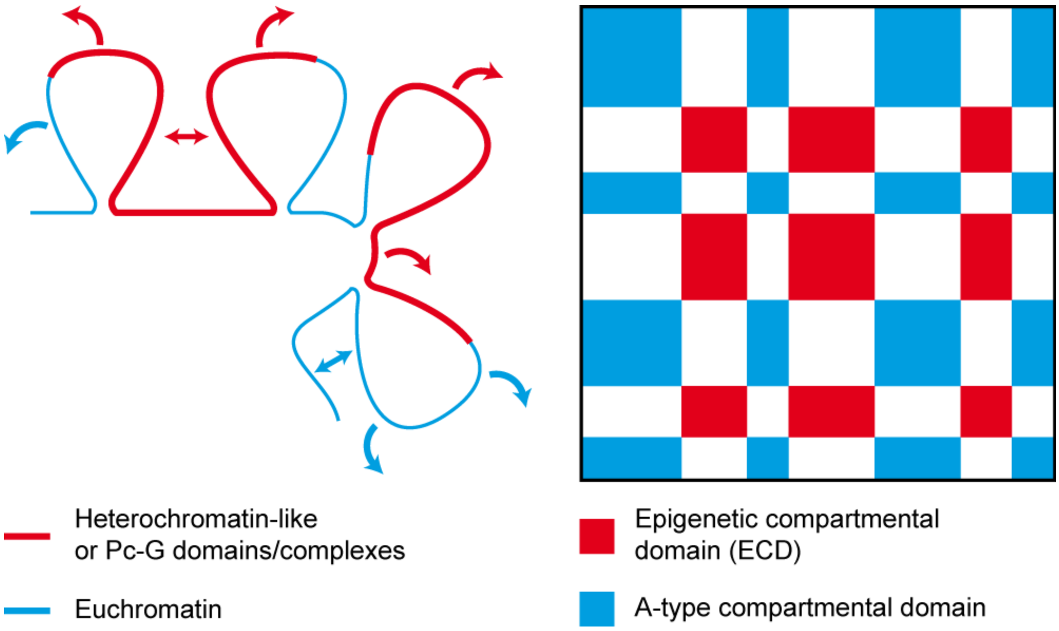


**Diagram C: *Cis-* and *trans-* contacts involving micro-phase separated heterochromatin-like or Pc-G domains/complexes generate ECDs.** Heterochromatin-*like* or Pc-G domains/complexes (red lines) segregate through like-with-like *cis-* (red double-headed arrows) and *trans-* (red arrows) interactions. These interactions generate contact enrichments that emerge as epigenetic compartmental domains (red EDCs in the cartoon Hi-C map). For euchromatin, *cis-* (blue double-headed arrows) and *trans-* (blue arrows) contacts generate the A-type compartmental domains (blue contact enrichments in cartoon Hi-C map). The cartoon Hi-C map was taken and modified from [4].

B-type compartmental domains are unlikely to be precisely equivalent to ECDs, *i.e.*, it is unlikely that B-type compartments are generated solely by contact enrichments that are a consequence of segregation of micro-phase separated heterochromatin-*like* and Pc-G domains/complexes. For example, it is known that B3 sub-compartment includes contact enrichments that result from interactions between lamin-associated loci [13]. HP1 proteins are also known to associate with the nuclear lamina [14] but the degree to which heterochromatin-*like* domains/complexes are involved in interactions between lamin-associated loci that generate the contact enrichments is not known. Functional experiments will be needed to define the precise extent to which ECDs overlap with B-type heterochromatic compartmental domains.

**Box S2.** The Flory-Huggins parameter ($\chi$) and the monomer as the "unit of incompatibility".

Polymers are very long molecules formed from small repeating units called monomers. Polymers come in many varieties but the standard types are formed from small hydrophobic hydrocarbon monomers (~100Da) that have low ionisation and polarization potential.

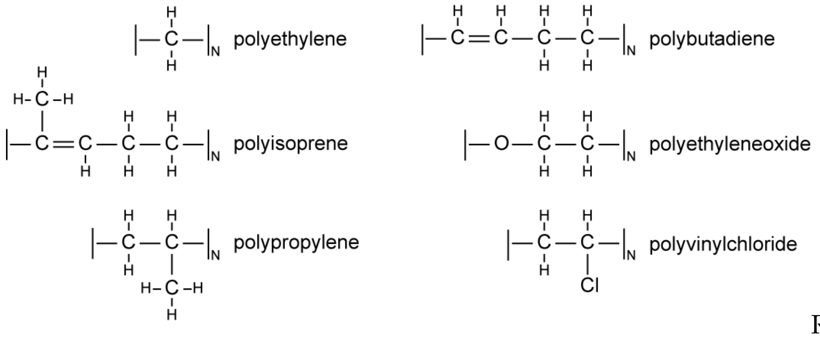


Redrawn from [15]

Homo-polymers formed from such monomers were the concern of Flory [16] and Huggins [17] when they set out to describe the thermodynamics of polymer mixing in simple solvents and, more importantly, the conditions under which they de-mix *i.e.*, phase separate into polymer-rich and solvent-rich phases. For such homo-polymers the tendency to phase separate is described by the Flory-Huggins parameter $\chi$ (Equation (S1) below), which quantifies the balance between the three types of interaction that take place when homo-polymers are added to a solvent, namely polymer-solvent, polymer-polymer and solvent-solvent interactions.

$$\chi_{\mathrm{ps}} = \frac{z}{k_{\mathrm{B}}T}\left[\varepsilon_{\mathrm{ps}} - \frac{1}{2}\left(\varepsilon_{\mathrm{pp}} + \varepsilon_{\mathrm{ss}}\right)\right] \qquad \text{(S1)}$$

These interactions can be modelled using mean-field lattice theory where polymer and solvent molecules arrange themselves randomly within in an infinite lattice structure of co-ordination number z (see below for two-dimensional lattice where z = 8), each occupying one lattice position. The lattice is set at the volume occupied by one monomer segment of the polymer; it is assumed that the lattice is incompressible so the volume of the solvent is equivalent to that of one monomer unit:

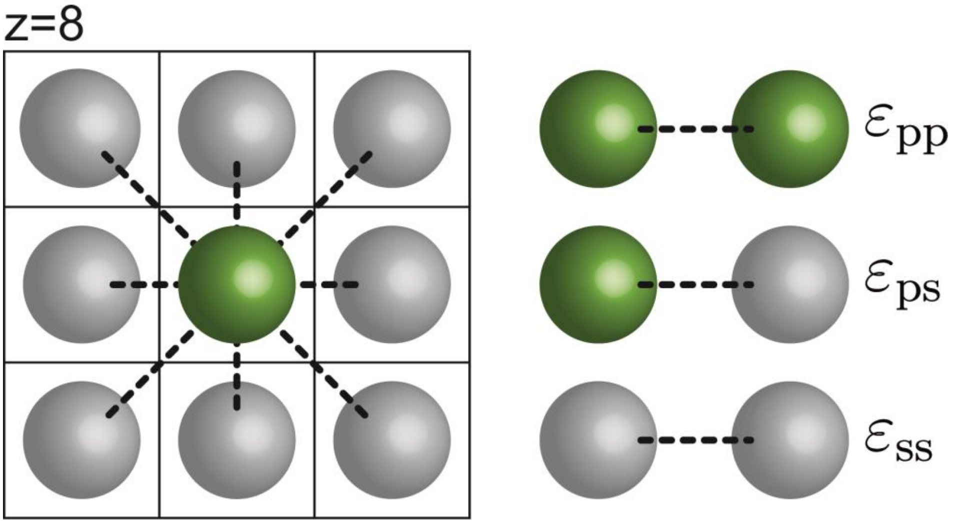


Taken and modified from [18]

.

Whether the polymer phase separates or not depends on the mean field energies per lattice site, *i.e.*, the energetic cost (in terms of thermal energy $k_BT$) of having pairs of polymer-solvent monomer units ($\varepsilon_{ps}$), pairs of polymer units ($\varepsilon_{pp}$) and pairs of solvent units ($\varepsilon_{ss}$) next to each other in the lattice. In the case of highly unfavourable interactions, where there is an energetic cost for having the polymer monomer site adjacent to the solvent site, monomers will prefer to be near each other (like-with-like) and phase separation is likely to occur (poor solvent; $\chi>0$). Above a particular value, $\chi_{crit}$, the critical value of the Flory-Huggins parameter where the energy of interaction overcomes the entropy of mixing, the polymer will collapse and phase separation is observed (see [18] for a more detailed discussion of the Flory-Huggins free energy of mixing equation). $\chi< 0$ in the case of highly favourable interactions between monomer and solvent, where monomers will avoid being near each other whereupon the polymer chain swells (good solvent). Thus $\chi$ is a measure of the degree of incompatibility of the polymer with the solvent and, as defined in Equation (S1), is inversely proportional to temperature. The sign of $\chi$ (+ve or –ve) is dependent upon the choice of monomer repeating unit; the *"unit of incompatibility"* for the Flory-Huggins approach to the miscibility of polymers is the *monomer*.

**Box S3.** The segregation product $\chi N$ and order-disorder (ODT) transition of BCPs.

The degree of micro-phase separation of bulk (undiluted) di-block copolymers (di-BCPs), where the A and B blocks are incompatible, is determined by the segregation product, $\chi N$, where N is the number of repeat units (monomers) that make up the polymer chain. Self-consistent field theory predicts that micro-phase separation takes place when $\chi N \approx 10.5$ [19], which has been confirmed experimentally [15]. When the di-BCP is symmetrical (volume fraction $f = 0.5$) and $\chi N > 10.5$ phase separation takes on the character of lamellae. The reason for this is that the covalent linkages stop the A and B blocks from macro-phase separating: the thermodynamic forces driving separation are counterbalanced by entropic forces from the covalent linkage. The resulting chain elasticity keeps the dissimilar A and B portions of the di-BCP apart. As a consequence, symmetric di-BCPs adopt extended configurations seen as lamellae that are in length scales similar to the molecular dimensions of the di-BCPs themselves (0.05-0.1μm).

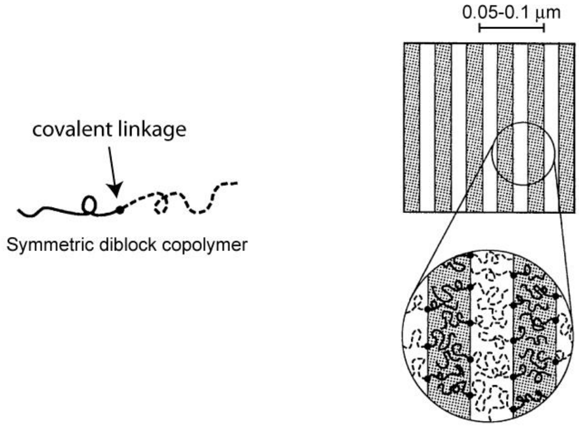


Taken from [20]

When $\chi N << 10$ for symmetric di-BCPs entropic factors dominate and they possess a homogeneous composition profile when plotting composition, $\phi_A$, versus the ensemble average of the bond vectors <r>. When $\chi$ or N are increased so that $\chi N \approx 10$ there are local fluctuations in composition owing to small variations in system entropy ($\sim N^{-1}$) or energy ($\sim \chi$) and this results in disordered states ($\chi N \leq 10$). As $\chi N$ is increased further there is an order-disorder transition (ODT; $\chi N_{ODT}$) where the disordered microstructure is replaced by a periodic lamellae mesophase ($\chi N \geq 10$) albeit weak A-B interactions still occur and, as consequence, the interfaces are weak and "wavy" (see $\phi_A$ vs $r_\perp$ for $\chi N \geq 10$).

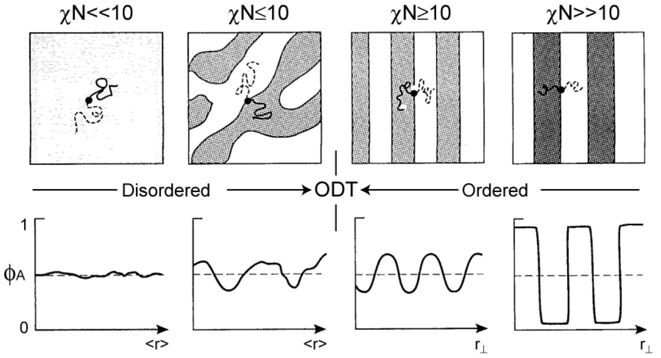


Taken from [20]

At the limit when $\chi N>>10$ energetic factors prevail and a strongly segregated lamellae pattern is observed where interfaces become narrow and micro-domains are composed of pure A or B and the ensemble bond vectors, r, are essentially perpendicular to the periodic mesophase. The evolution of the structures as the product $\chi N$ increases shows that phase separation close to the ODT ($\chi N \geq 10$) gives rise to interfaces that are weak, wavy, almost liquid-*like*, while much greater than the ODT ($\chi N>>10$) interfaces are narrow and sharp. The relationship between $\chi N$, $\chi N_{ODT}$ and degree of phase separation defined analytically with BCPs may provide insight into the character (degree) of the phase separation observed with heterochromatin-*like* domain/complexes compared to that seen with Pc-G domain/complexes. An important caveat is the domains/complexes are unlikely to behave like simple flexible chains governed by Gaussian statistics as assumed for bulk BCPs [15,19]. Because of this, heterochromatin-*like* and Pc-G domains/complexes in the interphase nucleus will have their own values for order-disorder transition, $\chi N_{ODT\_HC}$ and $\chi N_{ODT\_PC}$ respectively.

## References


1. Singh, P.B.; Newman, A.G. On the relations of phase separation and Hi-C maps to epigenetics. *R Soc Open Sci*. **2020**, *7*, 191976, doi:10.1098/rsos.191976.
2. Lieberman-Aiden, E.; van Berkum, N.L.; Williams, L.; Imakaev, M.; Ragoczy, T.; Telling, A.; Amit, I.; Lajoie, B.R.; Sabo, P.J.; Dorschner, M.O.; et al. Comprehensive mapping of long-range interactions reveals folding principles of the human genome. *Science* **2009**, *326*, 289–293, doi:10.1126/science.1181369.
3. Wang, S.; Su, J.H.; Beliveau, B.J.; Bintu, B.; Moffitt, J.R.; Wu, C.T.; Zhuang, X. Spatial organization of chromatin domains and compartments in single chromosomes. *Science* **2016**, *353*, 598–602, doi:10.1126/science.aaf8084.
4. Hildebrand EM.; Dekker, J. Mechanisms and Functions of Chromosome Compartmentalization. *Trends Biochem Sci*. **2020**, *45*, 385–396, doi:10.1016/j.tibs.2020.01.002.
5. Reinberg, D.; Vales, L.D. Chromatin domains rich in inheritance. *Science* **2018**, *361*, 33–34, doi:10. 1126/science.aat7871.
6. Illingworth, R.S.; Botting, C.H.; Grimes, G.R.; Bickmore, W.A.; Eskeland, R. PRC1 and PRC2 are not required for targeting of H2A.Z to developmental genes in embryonic stem cells. *PLoS One* **2012**, *7*, e34848. doi:10.1371/journal.pone.0034848.
7. Zheng, H.; Huang, B.; Zhang, B.; Xiang, Y.; Du, Z.; Xu, Q.; Li, Y.; Wang, Q.; Ma, J.; Peng, X.; et al. Resetting Epigenetic Memory by Reprogramming of Histone Modifications in Mammals. *Mol Cell*. **2016**, *63*, 1066–1079, doi:10.1016/j.molcel.2016.08.032.
8. Müller, M.M.; Fierz, B.; Bittova, L.; Liszczak, G.; Muir, T.W. A two-state activation mechanism controls the histone methyltransferase Suv39h1. *Nat. Chem. Biol*. **2016**, *12*, 188–193, doi:10.1038/nchembio.2008.
9. Kim, J.; Kingston, R.E. The CBX family of proteins in transcriptional repression and memory. *J. Biosci*. **2020**, *45*, 16.
10. Grau, D.J.; Chapman, B.A.; Garlick, J.D.; Borowsky, M.; Francis, N.J.; Kingston, R.E. Compaction of chromatin by diverse Polycomb group proteins requires localized regions of high charge. *Genes Dev*. **2011**, *25*, 2210–2221 doi:10.1101/gad.17288211.
11. Cheloufi, S.; Elling, U.; Hopfgartner, B.; Jung, Y.L.; Murn, J.; Ninova, M.; Hubmann, M.; Badeaux, A.I.; Euong Ang, C.; Tenen, D.; et al.; Abazova, N.; Hogue, M.; Tasdemir, N.; Brumbaugh, J.; Rathert, P.; Jude, J.; Ferrari, F.; Blanco, A.; Fellner, M.; Wenzel, D.; Zinner, M.; Vidal, S.E.; Bell, O.; Stadtfeld, M.; Chang, H.Y.; Almouzni, G.; Lowe, S.W.; Rinn, J.; Wernig, M.; Aravin, A.; Shi, Y.; Park, P.J.; Penninger, J.M.; Zuber, J.; Hochedlinger, K. The histone chaperone CAF-1 safeguards somatic cell identity. *Nature* **2015**, *528*, 218–224, (doi:10.1038/nature15749)
12. Borkent, M.; Bennett, B.D.; Lackford, B.; Bar-Nur, O.; Brumbaugh, J.; Wang, L.; Du, Y.; Fargo, D.C.; Apostolou, E.; Cheloufi, S.; et al. A serial shRNA screen for roadblocks to reprogramming identifies the protein modifier SUMO2. *Stem Cell Rep*. **2016**, *6*, 704–716, doi:10.1016/j.stemcr.2016.02.004.
13. Belaghzal, H.; Borrman, T.; Stephens, A.D.; Lafontaine, D.L.; Venev, S.V.; Weng, Z.; Marko, J.F.; Dekker, J. Compartment-dependent chromatin interaction dynamics revealed by liquid chromatin Hi-C. *bioRxiv*, **2019**, 704957. doi:10.1101/704957.
14. van Steensel, B.; Belmont, A.S. Lamina-Associated Domains: Links with Chromosome Architecture, Heterochromatin, and Gene Repression. *Cell* **2017**, *169*, 780–791, doi:10.1016/j.cell.2017.04.022.
15. Matsen, M.W. Self-consistent Field Theory and Its Applications. In. Soft Matter. Vol. 1: Polymer Melts and Mixtures. Eds. G. Gompper and M. Schick. Wiey verlag GmbH & Co. KGaA, Weinheim, **2006**.
16. Flory, P.J. Thermodynamics of High Polymer Solutions. *J. Chem. Phys*. **1941**, *9*, 660, doi.org/10.1063/1.1750971.
17. Huggins, M.L. Solutions of Long Chain Compounds. *J. Chem. Phys.* **1941**, *9*, 440, doi.org/10.1063/1.1750930.
18. Brangwynne, C.P.; Tompa, P.; Pappu, R.V. Polymer physics of intracellular phase transitions. *Nature Physics* **2015**, *11*, 899–904.
19. Leibler, L. Theory of Microphase Separation in Block Copolymers *Macromolecules* **1980**, *13*, 1602.
20. Bates, F.S. Polymer-polymer phase behavior. *Science* **1991**, *251*, 898–905, doi:10.1126/science.251.4996.898.

**Table S1.** Size range of heterochromatin-*like* complexes in man (H1 ES cells and 293T cells) and mouse (ES cells).

| | *H. sapiens* \| HP1α,HP1γ,H3K9me3\| H1 ES Cells | | *H. sapiens* \| HP1α,HP1β,H3K9me3\| 293T HEK Cells | | *M. musculus* \| HP1α,HP1β,HP1γ,H3K9me3 \| ES cells | |
|---|---|---|---|---|---|---|
| Size, kb | N | % | N | % | N | % |
| 100-90 | 30 | 0.16 | 229 | 0.71 | 223 | 2.18 |
| 90-80 | 24 | 0.13 | 328 | 1.02 | 271 | 2.65 |
| 80-70 | 35 | 0.19 | 477 | 1.48 | 364 | 3.56 |
| 70-60 | 85 | 0.45 | 667 | 2.07 | 566 | 5.53 |
| 60-50 | 154 | 0.82 | 1043 | 3.23 | 725 | 7.09 |
| 50-40 | 403 | 2.14 | 1845 | 5.71 | 1119 | 10.94 |
| 40-30 | 1118 | 5.93 | 3455 | 10.70 | 1583 | 15.48 |
| 30-20 | 3356 | 17.80 | 7014 | 21.72 | 2427 | 23.73 |
| 20-10 | 13648 | 72.39 | 17234 | 53.37 | 2949 | 28.84 |
| ***Total*** | ***18853*** | | ***32292*** | | ***10227*** | |
